# Investigating concordance in diabetes diagnosis between primary care charts (electronic medical records) and health administrative data: a retrospective cohort study

**DOI:** 10.1186/1472-6963-10-347

**Published:** 2010-12-23

**Authors:** Stewart B Harris, Richard H Glazier, Jordan W Tompkins, Andrew S Wilton, Vijaya Chevendra, Moira A Stewart, Amardeep Thind

**Affiliations:** 1 Centre for Studies in Family Medicine, Department of Family Medicine, Schulich School of Medicine & Dentistry, The University of Western Ontario, London, Ontario, Canada; 2 Institute for Clinical Evaluative Sciences, Toronto, Ontario, Canada; 3 Centre for Research on Inner City Health, in the Keenan Research Centre at the Li Ka Shing Knowledge Institute of St. Michael's Hospital, Toronto, Ontario, Canada; 4 Department of Family and Community Medicine, University of Toronto, Toronto, Ontario, Canada; 5 Department of Epidemiology & Biostatistics, Schulich School of Medicine & Dentistry, The University of Western Ontario, London, Ontario, Canada

## Abstract

**Background:**

Electronic medical records contain valuable clinical information not readily available elsewhere. Accordingly, they hold important potential for contributing to and enhancing chronic disease registries with the goal of improving chronic disease management; however a standard for diagnoses of conditions such as diabetes remains to be developed. The purpose of this study was to establish a validated electronic medical record definition for diabetes.

**Methods:**

We constructed a retrospective cohort using health administrative data from the Institute for Clinical Evaluative Sciences Ontario Diabetes Database linked with electronic medical records from the Deliver Primary Healthcare Information Project using data from 1 April 2006 - 31 March 2008 (N = 19,443). We systematically examined eight definitions for diabetes diagnosis, both established and proposed.

**Results:**

The definition that identified the highest number of patients with diabetes (N = 2,180) while limiting to those with the highest probability of having diabetes was: individuals with ≥2 abnormal plasma glucose tests, or diabetes on the problem list, or insulin prescription, or ≥2 oral anti-diabetic agents, or HbA1c ≥6.5%. Compared to the Ontario Diabetes Database, this definition identified 13% more patients while maintaining good sensitivity (75%) and specificity (98%).

**Conclusions:**

This study establishes the feasibility of developing an electronic medical record standard definition of diabetes and validates an algorithm for use in this context. While the algorithm may need to be tailored to fit available data in different electronic medical records, it contributes to the establishment of validated disease registries with the goal of enhancing research, and enabling quality improvement in clinical care and patient self-management.

## Background

Diabetes is a significant and costly disease that is projected to affect 438 million individuals worldwide by the year 2030 [1]. The burden of diabetes lies not only in the absolute number of individuals with the disease, but in the severe complications associated with unmanaged diabetes. In Canada, it is estimated that 3-5% of the population may have undiagnosed diabetes [2,3], and Ontario has seen minimal progress in reducing the rates of complications due to the disease [4]. Therefore, a critical need exists in our healthcare system to efficiently diagnose and effectively manage patients with diabetes. Lack of available data systems for clinical use and quality improvement is a major barrier to achieving this goal.

There is a significant effort in Canada and the United States towards the uptake of electronic medical records (EMRs) in an effort to obtain the benefits of a computerized health care system [5]. EMRs are designed to increase the ease and efficiency of medical practice, and past research has demonstrated the benefits of pairing an electronic medical system with disease registries [6]. Diabetes registries offer users a quick and efficient way to identify high-risk patients and measure clinical outcomes, with potential to improve care both at the physician level with adherence to clinical practice guidelines, and the patient level, with self-management and improved medication adherence. However, the ability to accurately identify those individuals diagnosed with the disease remains a critical feature of any successful chronic disease registry, and a crucial first step for converting an EMR into a researchable database to measure and address quality of care [7,8]. Currently, many jurisdictions in North America lack both wide adoption of EMRs and validated chronic disease registries. One approach to understanding the prevalence and burden of disease due to diabetes has been the use of the United States Department of Veterans Affairs, or Canada's National Diabetes Surveillance System (NDSS), both based on administrative claims [9,10]. The Ontario, Canada version of this approach is known as the Ontario Diabetes Database (ODD), a de-identified listing of those with diabetes among all those eligible for health care in this universal health insurance system. The ODD excludes people with gestational diabetes and does not distinguish between type 1 and type 2 diabetes. Based on a validated algorithm against primary care chart data in 1998, the ODD exhibited a high level of sensitivity and specificity (86 percent and 97 percent, respectively) with a positive predictive value of 80%, and thus has been a valuable tool for surveillance purposes [11]. However, administrative data often lack clinical details such as test results and medications, limiting their usefulness for improving clinical care.

EMR data generally contain rich clinical detail and therefore hold the promise of being used to understand and improve processes of care and their outcomes. A validated EMR approach to identify people with diabetes is therefore needed. The primary objective of this research was to assess the feasibility and performance of an EMR definition of diabetes by developing EMR approaches to defining diabetes and comparing these approaches to the ODD.

## Methods

### Study Database and Population

A retrospective cohort was constructed using the ODD linked with EMR data from the Deliver Primary Healthcare Information (DELPHI) Project [12]. The target practice population of EMR data included all practices enrolled in the DELPHI Project. The DELPHI database is based at the Centre for Studies in Family Medicine at The University of Western Ontario and is a researchable database established in 2005. The database is populated with de-identified electronic health records of 22 family practitioners and their patients in Southwestern Ontario, including both rural and urban practices, and is inclusive of all patients in the practice. The DELPHI database consists of patient medical records entered into HealthScreen™, one of the electronic medical record software programs used in Ontario. The database was created by pooling the de-identified health records of patients using data from 1 April 2006 - 31 March 2008. Using a common unique encrypted identifier, a data linkage was performed between patients enrolled in the DELPHI database prior to April 1, 2006 and the ODD and Ontario's health registry, the Registered Persons Database, both held at the Institute for Clinical Evaluative Sciences (N = 19,443).

### Diabetes Definitions

A detailed description of each of the eight diabetes definitions is found in Table 1. A Base Definition (Definition 1) was selected based on its alignment with the Canadian 2003 Clinical Practice Guideline (CPG) diagnostic glycemic values and types of medication prescribed [13]. From the Base Definition, a series of five permutations (Definition 1.a. - 1.e.) were selected to determine the most comprehensive EMR definition of diabetes achievable. These permutations were systematically examined to determine the impact of the number of oral anti-diabetic drugs prescribed and the number of plasma glucose tests, as well as the clinically important items of 'diabetes on problem list' and HbA1c ≥6.5%. 'Diabetes on problem list' is consistently used in the literature for capturing patients with diabetes [14] and was manually reviewed by an expert source (one of the co-authors; SH) to ensure coding accuracy and reliability of diagnosis. Patients with gestational diabetes, prediabetes and polycystic ovary syndrome (PCOS) were excluded during the manual coding process. The HbA1c value of 6.5% was recently approved by the American Diabetes Association as a screening test by an international committee of experts [15-19]. The remaining two diabetes definitions included the definition proposed by the Ontario Ministry of Health and Long-Term Care in Ontario for the Baseline Diabetes Database Initiative (BDDI) to populate a provincial diabetes registry (Definition 2), and the previously validated ODD definition (Definition 3).

**Table 1:** Number of patients diagnosed with diabetes (true positives between linked health administrative-EMR dataset) and associated crude prevalence rates categorized by diabetes definition

Definition	# Patients with Diabetes	Crude Prevalence %
**1. Base Definition: ≥2 plasma glucose tests^a ^*OR *insulin *OR *≥2 oral anti-diabetic drugs**	975	5.0
**a. Base *OR *HbA1c ≥6.5%**	1515	7.8
**b. Base *OR *diabetes on problem list**	1905	9.8
**c. Base *OR *HbA1c ≥6.5% *OR *diabetes on problem list**	2180	11.2
**d. Base *OR *HbA1c ≥6.5% *OR *diabetes on problem list; Limit base definition to ≥1 oral anti-diabetic drugs**	2329	12.0
**e. Base *OR *HbA1c ≥6.5% *OR *diabetes on problem list; Limit base definition to ≥1 oral anti-diabetic drugs *OR *≥1 plasma glucose tests**	2329	12.0
**2. Registry: ≥2- ICD 250 codes *AND *≥1 HbA1c lab value**	1586	8.2
**3. ODD: one hospitalization discharge with a diagnostic code for diabetes (ICD9 250) *OR *two physician service claims with a diagnosis recorded for diabetes (ICD8 250) within a 2-year period**	1935	10.0

^a^Plasma glucose tests include: fasting plasma glucose ≥ 7.0 mmol/L; casual plasma glucose ≥ 11.1 mmol/L; 2-hour plasma glucose in a 75-g oral glucose tolerance test ≥ 11.1 mmol/L

### Analysis

To investigate variation in diabetes diagnosis, an analysis using 2 × 2 factorials was conducted using the eight varying definitions of diabetes. Sensitivity, specificity, positive predictive value (PPV) and negative predictive value (NPV) were calculated for each 2 × 2 factorial. This conventional approach is used when comparing a "gold standard" condition to a test outcome, such that each 2 × 2 factorial would assign one definition as the "gold standard" and one definition as the test outcome. Sensitivity and specificity were calculated as the proportion of patients with diabetes identified by a test definition who either had or did not have diabetes according to the comparator "gold standard". PPV and NPV were calculated as the proportion of patients diagnosed with or without diabetes identified by the test definition and confirmed by the comparator "gold standard" definition. Prevalence estimates were calculated according to the number of patients with diabetes identified by each definition. Statistical analyses were performed using SAS 9.2 [20].

Assuming a prevalence of diabetes of 8.8% [21] and based on the size of the patient population in the linked EMR dataset, reasonably precise estimates of sensitivity and specificity were ensured. For example, with 19,443 people in the dataset, 1,710 would be expected to have diabetes and for an expected sensitivity of 80%, the 95% confidence interval would be ± 1.9%. For an expected specificity of 95%, the 95% confidence interval would be ± 0.3%. Our sample size was substantially larger than the approximately 3000 charts used in an office-based validation of an administrative data algorithm for diabetes, and therefore suitable for the purposes of this research [11].

### Ethical Approval

Ethical approval for this study was obtained through the ICES/Sunnybrook research ethics review as part of the standard process. Ethical approval at The University of Western Ontario was included under the ethically approved umbrella of the DELPHI Project.

## Results

The linked health administrative-EMR dataset included 22 family practitioners and 19,443 patients in Southwestern Ontario. Physician demographics are presented in Table 2. Physician characteristics were similar except that DELPHI physicians were more likely to be based in a rural area.

**Table 2:** Demographics of 22 DELPHI physicians compared to physicians in the province of Ontario

	DELPHI	Ontario^a^
	N = 22	N = 11,819
**Male (%)**	68.0	61.7
**Years since graduation**	26.5	23.5
**Rural (%)**	55.0	10.1

**^a^**Institute for Clinical Evaluative Sciences, August 2008

Table 1 displays the diagnosed population by diabetes definition. Crude prevalence rates varied by definition, from 5.0% (Base Definition 1) to 12.0% (Definition 1.d./1.e.).

Additional file 1 reports the sensitivity and specificity analysis by diabetes definitions. The definition that identified the highest number of patients with diabetes while at the same time limiting to those with the highest probability of having diabetes was Definition 1.c. (N = 2,180). This definition identified 13% (N = 245) more patients than the ODD definition while maintaining good sensitivity (75%) and specificity (98%) in comparison with the ODD.

Both Definitions 1.d. and 1.e. (N = 2,329) identified the highest number of patients with diabetes using a broader definition, and identified 20% (N = 394) more patients than the ODD definition while maintaining good sensitivity (1.d. = 73%; 1.e. = 72%) and specificity (1.d./1.e. = 99%) in comparison with the ODD.

The currently proposed diabetes definition for use in the BDDI to pre-populate a diabetes registry missed 27% (N = 594) of those identified as having diabetes when compared to our most comprehensive definition (Definition 1.c.); however it held good sensitivity (97%) and specificity (98%) in comparison with the ODD.

Patient demographics are presented in Table 3. Among patients in the linked dataset, 11.2% were found to have diabetes using Definition 1.c. Patients with diabetes were slightly older and were much more likely to have coronary artery disease, hypertension, and a positive family history of diabetes.

**Table 3:** Characteristics of 19442a patients identified in the EMR database between 1 April 2006 - 31 March 2008, stratified by diabetes diagnosis and sex

	Overall			Men		Women	
**% (unless otherwise indicated)**	**General** **N = 19442**^**a**^	**DM**^**b**^ **N = 2180**	**Non-DM N = 17262**	**DM**^**b**^ **N = 1174**	**Non-DM** **N = 7868**	**DM**^**b**^ **N = 1006**	**Non-DM** **N = 9394**

**Males**	**46.5**	53.9	45.6				
**Age **(Mean)	**42.5**	45.0	42.5				
**Rurality**	**44.0**	46.1	43.7	46.4	43.3	45.7	44.1
**New Immigrant**^c^	**0.4**	0.6	0.4	0.7	0.5	0.0	0.4
**Income**							
**Quintile 1-low**	**9.7**	10.9	9.5	10.4	9.3	11.5	9.7
**Quintile 5-high**	**32.0**	30.6	32.2	30.3	32.2	31.0	32.1
**Diagnoses**							
**Diabetes on problem list**	**8.5**	75.8	0.0	73.3	0.0	78.7	0.0
**Diabetes complication problem list**^d^	**25.5**	61.7	20.9	61.6	21.4	61.7	20.5
**Polycystic ovary syndrome (PCOS)**	**0.1**	0.0	0.1	0.0	0.0	0.4	0.2
**Coronary Artery Disease (CAD)**	**4.7**	14.0	3.5	16.7	4.6	11.0	2.6
**Hypertension**	**16.5**	43.0	13.2	42.2	12.3	44.0	13.9
**Family History of Diabetes**	**6.8**	14.2	5.9	9.9	4.2	19.2	7.2
**Visits to Family Physician **(Mean)	**8.4**	11.5	7.9	10.7	7.2	12.4	8.4
**Prescribed Diabetes Medications**^e^							
**Insulin Ever**	**1.7**	15.4	0.0	16.1	0.0	14.6	0.0
**Metformin Ever**	**6.6**	52.9	0.7	53.9	0. 8	51.8	0.6
**Oral hypoglycemic agents**	**4.7**	40.4	0.1	42.3	0.0	38.1	0.2

^a^Demographics missing for 1 patient; ^b^Diabetes diagnosis using Definition 1.c.

^c^New immigrant - patients not eligible for provincial health insurance (OHIP) as of the study start date used as proxy to determine new immigrant status; ^d^Diabetes related complication including: coronary artery disease, hypertension, chronic obstructive pulmonary disease, polycystic ovarian syndrome, dyslipidemia, stroke/transient ischemic attack, erectile dysfunction, cardiovascular disease; ^e^Over a 2 year time period

## Discussion

Current literature suggests that large administrative datasets lack precision and detail [14,22-25], and as the clinical practice world progresses toward electronic medical systems and chronic disease registries, there is a clear need to validate a methodology for identifying individuals with chronic diseases and specifically, diabetes. Our results support the ODD algorithm for diabetes diagnosis in administrative data, and suggest an EMR standard for diagnosis of disease in the DELPHI database using Definition 1.c. This definition clearly aligns with the Canadian 2003 clinical practice guideline diagnostic criteria as it incorporates two or more abnormal plasma glucose tests, or the use of insulin, or two or more oral antidiabetic drugs [13]. It also aligns with clinically significant items including 'diabetes on the problem list' [14] or having an HbA1c ≥6.5%. This research supports the incorporation of HbA1c ≥6.5%, a value recently approved by the American Diabetes Association to diagnose [15-19,26].

This research tested varying definitions in the assessment of an EMR standard for diabetes diagnosis, some established (for example, the Ontario Diabetes Database definition) and some proposed (for example, the BDDI established by the Ontario Ministry of Health and Long-Term Care). We started with a Base Definition, and systematically examined a series of permutations to determine the most comprehensive EMR definition of diabetes achievable. The EMR standard definition created for this study identified more patients than the ODD definition while maintaining good sensitivity and specificity in comparison with the ODD, and represented the most comprehensive EMR definition of diabetes achievable in our data. Our findings also support Definition 1.d. as an alternative EMR diabetes diagnosis, representing a 7% increase from our EMR standard. Clinically, patients on one or more oral anti-diabetic agents (when compared to two or more in our EMR standard) could have polycystic ovarian syndrome (PCOS) or pre-diabetes; therefore Definition 1.d. should only be used when the critical first step is taken of reliably eliminating patients with these conditions. This definition can be recommended in EMRs that reliably identify PCOS and pre-diabetes or when these conditions can otherwise be excluded.

Health administrative and EMR data serve as valuable tools in the ongoing surveillance of disease, with EMRs offering an added advantage of patient level process and outcome measures. Furthermore, EMRs have the potential of identifying unbiased diabetes incident cases, a measure that can be challenging when using administrative data solely as the data source [22,27]. Improving case identification using administrative or EMR datasets points to the need for continued linkages between these types of databases, with a goal to improving the performance of both and establishing validated disease registries [22,28].

The majority of Canadians with diabetes are managed in the family practice setting [29], and the process of populating a diabetes registry through the adoption of an EMR provides access to a valuable resource of clinical information that is not readily available elsewhere. Diabetes registries offer users a quick and efficient way to identify high-risk patients and measure clinical outcomes, two critical first steps in the ongoing surveillance of diabetes for clinicians, researchers and policy makers; however the ability to accurately identify those individuals diagnosed with the disease remains a critical feature of any successful chronic disease registry [7,8].

It is important to acknowledge a number of limitations in this research. Firstly, the DELPHI dataset does not include consult letters or visit notes, valuable determinants of diagnosis used in previous literature. Although it is not possible to assess false positives in the dataset, this method does shed light on the proportion of people with diabetes missing. A second limitation is that the DELPHI dataset is not population-based; however it does represent all patients from a variety of different types of practices and settings throughout Southwestern Ontario that do not differ significantly from Ontario physicians on the whole (with the exception of rurality identified in the results). Our findings reveal slightly higher diabetes rates in the DELPHI population (11.2% crude prevalence rate using our EMR standard; 10.0% crude prevalence rate using ODD Definition) when compared to the most recent ICES 2004/05 diabetes rate for Southwestern Ontario of 7.8% [30] or Ontario rate of 8.8% [21]; however this is a population receiving active medical care so rates are likely higher when compared to the general population. A third limitation to this research that is inherent in both health administrative data and EMRs is a bias towards individuals who use health services; therefore, our dataset would only include those individuals receiving medical care. Furthermore, patients who migrate between clinicians may contribute to under-reporting because no one clinician record would be representative of the delivery of care to those patients. Lastly, based on the limitations of provincial billing codes, the ODD cannot distinguish between type 1 and type 2 diabetes mellitus. Type 2 diabetes accounts for 90-95% of the diagnosed population and therefore we are confident that the results of this study can be applied for determining type 2 diabetes diagnosis in the adult population [31].

## Conclusions

This research investigated the concordance in diabetes diagnosis between health administrative and EMR databases as a first step in developing and validating an EMR definition of diabetes. This study establishes a new EMR standard definition of diabetes (individuals with ≥2 abnormal plasma glucose tests, or diabetes on the problem list, or insulin prescription, or ≥2 oral anti-diabetic agents, or HbA1c ≥6.5%) and provides an alternative definition, depending on the completeness of EMR diagnoses. While this definition should be tested in other EMR systems, it is a promising first step in using EMR-based data to improve diabetes care. Future pursuit of a diabetes registry will benefit from a comprehensive understanding of the challenges of diabetes diagnosis using electronic medical records, and holds important potential for contributing to and enhancing chronic disease registries with the goal of improving chronic disease management and health outcomes.

## List of abbreviations

BDDI: Baseline diabetes dataset initiative; CPG: Clinical practice guidelines; DELPHI: Delivering primary healthcare information; DM: diabetes mellitus; EMR: Electronic medical record; HA: health administrative; ICES: Institute for clinical evaluative sciences; NDSS: National diabetes surveillance system; NPV: Negative predictive value; OAD: Oral antidiabetic drug; ODD: Ontario diabetes database; OLIS: Ontario laboratories information system; OGTT: oral glucose tolerance test; PCOS: polycystic ovary syndrome; PPV: Positive predictive value.

## Competing interests

The authors declare that they have no competing interests.

## Authors' contributions

The study was conceived by RG, SH, JT, VC, MS and AT, designed by RG, SH, JT and AW, data analysis was performed by AW, data interpretation was done primarily by RG, SH, JT and AW, and drafting of the paper was done jointly by RG and JT with substantial input from SH. All authors revised the paper and approved the final version.

## Pre-publication history

The pre-publication history for this paper can be accessed here:

http://www.biomedcentral.com/1472-6963/10/347/prepub

## Supplementary Material

Additional file 1 **Validation results for identifying patients with diabetes using a linked health administrative-EMR dataset, between 1 April 2006 - 31 March 2008**. Sensitivity, specificity, positive predictive value, negative predictive value and kappa results for identifying patients with diabetes based on 2 × 2 factorial analysis of 8 varying diagnosis definitions. Click here for file
