# Supplementary material for: Investigating concordance in diabetes diagnosis between primary care charts (electronic medical records) and health administrative data: a retrospective cohort study
Source: BMC Health Serv Res. 2010 Dec 23;10:347. doi: 10.1186/1472-6963-10-347 (PMC3022877; doi:10.1186/1472-6963-10-347)
Supplement: Additional file 1 — Validation results for identifying patients with diabetes using a linked health administrative-EMR dataset, between 1 April 2006 - 31 March 2008. Sensitivity, specificity, positive predictive value, negative predictive value and kappa results for identifying patients with diabetes based on 2 × 2 factorial analysis of 8 varying diagnosis definitions. [file 1472-6963-10-347-S1.DOC]

## Additional File 1 - Validation results for identifying patients with diabetes using a linked health administrative-EMR dataset, between 1 April 2006 – 31 March 2008

| **Definition** | | **TP^a^** | **TN^b^** | **FN^c^** | **FP^d^** | **Sensitivity** | **Specificity** | **PPV^e^** | **NPV^f^** | **Kappa** |
| --- | --- | --- | --- | --- | --- | --- | --- | --- | --- | --- |
| **1. Base vs.** | **1.a. Base or HbA1c** | 975 | 17928 | 0 | 540 | 100 | 97.1 | 64.4 | 100 | 0.7690 |
|  | **1.b. Base or PL^g^** | 975 | 17538 | 0 | 930 | 100 | 95.0 | 51.2 | 100 | 0.6541 |
|  | **1.d. Base-1OAD^h^ or HbA1c or PL** | 975 | 17114 | 0 | 1354 | 100 | 92.7 | 41.9 | 100 | 0.5590 |
|  | **1.e. Base-1OAD-1PGT^i^ or HbA1c or PL** | 975 | 17114 | 0 | 1354 | 100 | 92.7 | 41.9 | 100 | 0.5590 |
|  | **2. Registry** | 793 | 17857 | 182 | 793 | 81.3 | 95.7 | 50.0 | 99.0 | 0.5941 |
|  | **3. ODD** | 888 | 17421 | 87 | 1047 | 91.1 | 94.3 | 45.9 | 99.5 | 0.5825 |
| **1.a. Base or HbA1c vs.** | **1.b. Base or PL** | 1240 | 17263 | 275 | 665 | 81.8 | 96.3 | 65.1 | 98.4 | 0.6990 |
|  | **1.d. Base-1OAD or HbA1c or PL** | 1515 | 17114 | 0 | 814 | 100 | 95.5 | 65.1 | 100 | 0.7662 |
|  | **1.e. Base-1OAD-1PGT or HbA1c or PL** | 1515 | 17114 | 0 | 814 | 100 | 95.5 | 65.1 | 100 | 0.7662 |
|  | **2. Registry** | 1138 | 17480 | 377 | 448 | 75.1 | 97.5 | 71.8 | 97.9 | 0.7109 |
|  | **3. ODD** | 1312 | 17305 | 203 | 623 | 86.6 | 96.5 | 67.8 | 98.8 | 0.7374 |
| **1.b. Base or PL vs.** | **1.d. Base-1OAD or HbA1c or PL** | 1905 | 17114 | 0 | 424 | 100 | 97.6 | 81.8 | 100 | 0.8878 |
|  | **1.e. Base-1OAD-1PGT or HbA1c or PL** | 1905 | 17114 | 0 | 424 | 100 | 97.6 | 81.8 | 100 | 0.8878 |
|  | **2. Registry** | 1281 | 17233 | 624 | 305 | 67.2 | 98.3 | 80.8 | 96.5 | 0.7079 |
|  | **3. ODD** | 1443 | 17046 | 462 | 492 | 75.8 | 97.2 | 74.6 | 97.4 | 0.7243 |
| **1.c. Base or HbA1c or PL vs.** | **1. Base** | 975 | 17263 | 1205 | 0 | 44.7 | 100 | 100 | 93.5 | 0.5896 |
|  | **1.a. Base or HbA1c** | 1515 | 17263 | 665 | 0 | 69.5 | 100 | 100 | 96.3 | 0.8018 |
|  | **1.b. Base or PL** | 1905 | 17263 | 275 | 0 | 87.4 | 100 | 100 | 98.4 | 0.9248 |
|  | **1.d. Base-1OAD or HbA1c or PL** | 2180 | 17114 | 0 | 149 | 100 | 99.1 | 93.6 | 100 | 0.9626 |
|  | **1.e. Base-1OAD-1PGT or HbA1c or PL** | 2180 | 17114 | 0 | 149 | 100 | 99.1 | 93.6 | 100 | 0.9626 |
|  | **2. Registry** | 1414 | 17091 | 766 | 172 | 64.9 | 99.0 | 89.2 | 95.7 | 0.7250 |
|  | **3. ODD** | 1632 | 16960 | 548 | 303 | 74.8 | 98.2 | 84.3 | 96.9 | 0.7688 |
| **1.d. Base-1OAD or HbA1c or PL vs.** | **1.e. Base-1OAD-1PGT or HbA1c or PL** | 2200 | 16985 | 129 | 129 | 94.5 | 99.3 | 94.5 | 99.3 | 0.9371 |
|  | **2. Registry** | 1461 | 16989 | 868 | 125 | 62.7 | 99.3 | 62.7 | 95.1 | 0.7191 |
|  | **3. ODD** | 1707 | 16886 | 622 | 228 | 73.3 | 98.7 | 88.2 | 96.5 | 0.763 |
| **1.e. Base-1OAD-1PGT or HbA1c or PL vs.** | **2. Registry** | 1441 | 16969 | 888 | 145 | 61.9 | 99.2 | 90.9 | 95.0 | 0.7078 |
|  | **3. ODD** | 1676 | 16855 | 653 | 259 | 72.0 | 98.5 | 86.6 | 96.3 | 0.7600 |
| **2. Registry vs. 3. ODD** |  | 1545 | 17467 | 41 | 390 | 97.4 | 97.8 | 79.8 | 99.8 | 0.8655 |

^a^TP – True Positive; ^b^TN – True Negative; ^c^FN – False Negative; ^d^FP – False Positive; ^e^PPV – Positive Predictive Value; ^f^NPV – Negative Predictive Value;

^g^PL – Problem List; ^h^1OAD – 1 or more oral anti-diabetic drugs; ^i^1PGT – 1 or more plasma glucose tests
